# Supplementary material for: Kcnq (Kv7) channels exhibit frequency-dependent responses via partial inductor-like gating dynamics
Source: Commun Biol. 2025 Jun 5;8:866. doi: 10.1038/s42003-025-08302-6 (PMC12141596; doi:10.1038/s42003-025-08302-6)
Supplement: Supplementary file 1 — Supplementary information [file 42003_2025_8302_MOESM1_ESM.pdf]

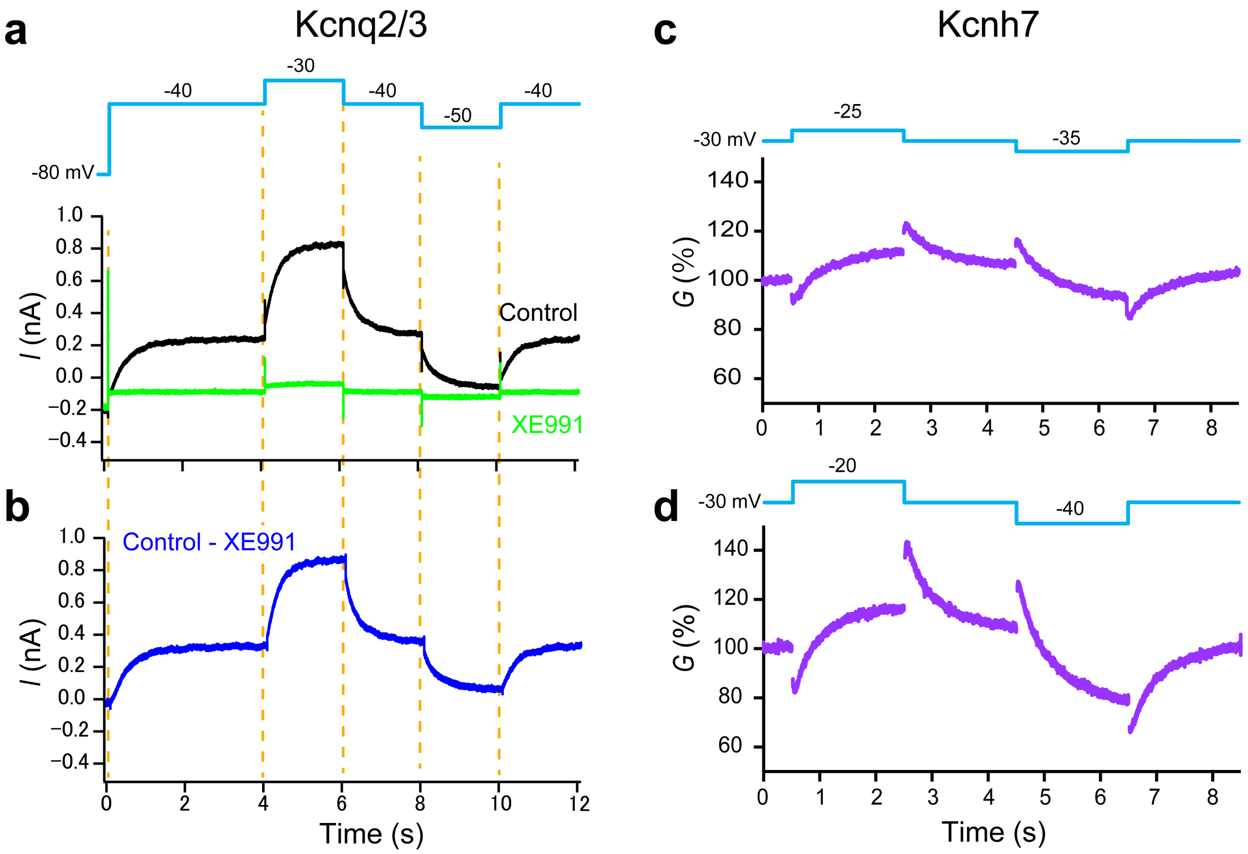

Supplementary Figure 1 Calculation of current responses to square voltage steps

a) (upper) Voltage protocols applied to *Kcnq2/3*-expressing cells. (lower) Representative traces recorded in the presence (green) or absence (black) of XE991 (20 µM). **b)** The representative Kcnq2/3 current calculated from data in a. The Kcnq2/3 current was calculated by subtracting currents recorded in the presence of XE991 from that in the control external solution. **c, d)** Representative Kcnh7 conductance relative to the baseline in response to ±5 mV (c) or ±10 mV (d) voltage changes.

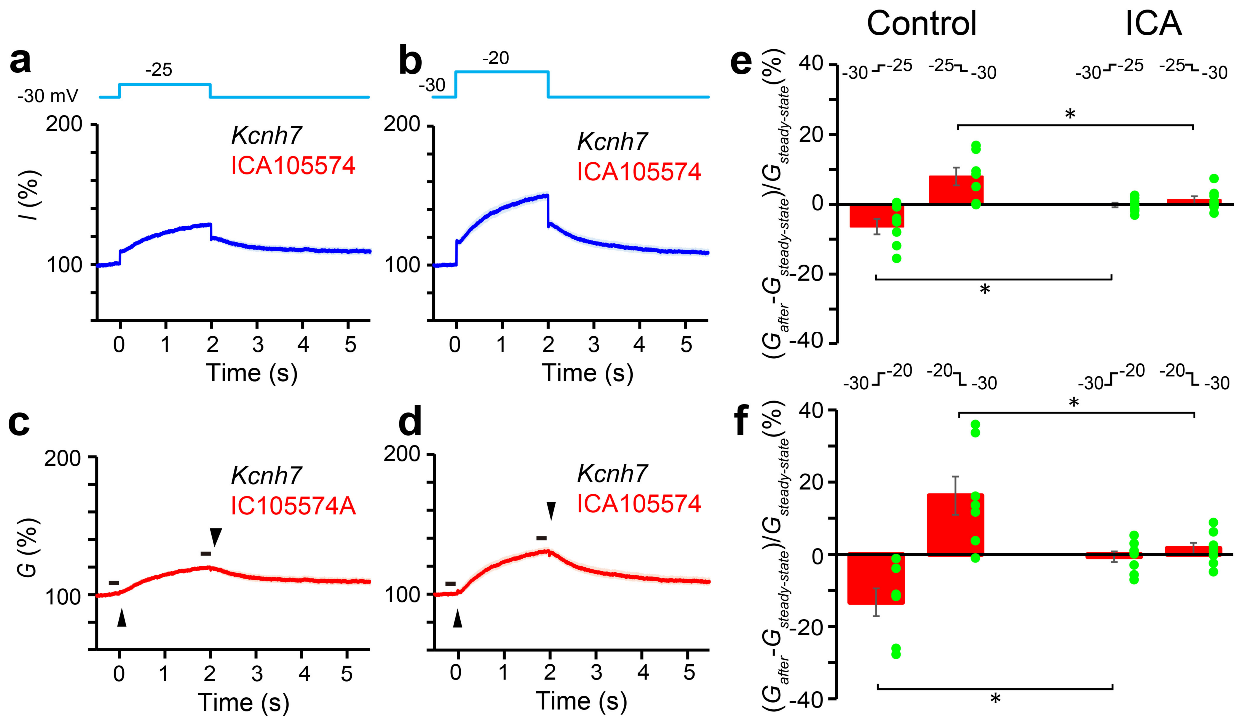

Supplementary Figure 2 Rapid inactivation and recovery from it is essential for the opposing conductance change

a, b) Voltage protocols applied to *Kcnh7*-expressing cells (upper traces) and average Kcnh7 currents relative to baseline (lower traces) in the presence of ICA105574 (2 μM) in response to depolarizing voltage steps of 5 mV (a) and 10 mV (b) (n = 8). Shaded areas indicate the mean ± SEM. **c, d)** The average Kcnh7 conductance relative to the baseline in the presence of ICA105574 in response to depolarizing voltage steps of 5 mV (c) and 10 mV (d). Shaded areas indicate the mean ± SEM. **e, f)** Magnitudes of opposing conductance changes (arrowheads in c and d) relative to the preceding steady-state conductance (black bars in c and d) in the presence (right, ICA, n = 8) or absence (left, control, n = 7) of ICA105574 in response to depolarizing voltage steps of 5 mV (e) and 10 mV (f). The data in the absence of ICA105574 (control) were same with those presented in Fig. 3s, t. Green dots represent individual data and red bars represent the mean ± SEM. *P < 0.05 (Welch's t-test).

Supplementary Table 1. Parameters of KCNQ channel kinetics

θ_m	-61	(mV)
k_m	19.5	(mV)
τ_{m0}	20	(ms)
τ_{m1}	400	(ms)
φ_m	-61	(mV)
σ_{m0}	35	(mV)
σ_{m1}	-25	(mV)
h_0	0.3	
θ_h	-30	(mV)
k_h	-15	(mV)
τ_{h0}	1	(ms)
τ_{h1}	10	(ms)
φ_h	-30	(ms)
σ_{h0}	6	(mV)
σ_{s1}	-10	(mV)

Supplementary Table 2. Half-conductance potential (V_{half}) and slope factor (k) in Fig. 7d

	V_{half} (mV)			k (mV)			n
Wild-type	-11.1 ± 1.0	*** (Wild-type vs R213Q)	*** (Wild-type vs R144Q)	10.5 ± 0.3	* (Wild-type vs R213Q)	*** (Wild-type vs R144Q)	12
R213Q	9.4 ± 1.9	*** (R213Q vs R144Q)		12. 2 ± 0.5	* (R213Q vs R144Q)		13
R144Q	-23.9 ± 1.3			14.2 ± 0.7			14

***$P < 0.001$, *$P < 0.05$ (one-way ANOVA; post-hoc Holm–Šidák test).
