# Supplementary material for: Kcnq (Kv7) channels exhibit frequency-dependent responses via partial inductor-like gating dynamics
Source: Commun Biol. 2025 Jun 5;8:866. doi: 10.1038/s42003-025-08302-6 (PMC12141596; doi:10.1038/s42003-025-08302-6)
Supplement: Supplementary file 2 — Description of Additional Supplementary Files [file 42003_2025_8302_MOESM2_ESM.docx]

**Description of Additional Supplementary Files**

File name- Supplementary Data 1

File description- The source data behind the graphs in the paper.
