# Supplementary material for: Kcnq (Kv7) channels exhibit frequency-dependent responses via partial inductor-like gating dynamics
Source: Commun Biol. 2025 Jun 5;8:866. doi: 10.1038/s42003-025-08302-6 (PMC12141596; doi:10.1038/s42003-025-08302-6)
Supplement: Supplementary file 4 — Reporting Summary [file 42003_2025_8302_MOESM4_ESM.pdf]

nature portfolio

Corresponding author(s): Kouichi Hashimoto

Last updated by author(s): May 14, 2025

Reporting Summary

Nature Portfolio wishes to improve the reproducibility of the work that we publish. This form provides structure for consistency and transparency in reporting. For further information on Nature Portfolio policies, see our Editorial Policies and the Editorial Policy Checklist.

Statistics

For all statistical analyses, confirm that the following items are present in the figure legend, table legend, main text, or Methods section.

n/a	Confirmed	
☐	☒	The exact sample size (*n*) for each experimental group/condition, given as a discrete number and unit of measurement
☐	☒	A statement on whether measurements were taken from distinct samples or whether the same sample was measured repeatedly
☐	☒	The statistical test(s) used AND whether they are one- or two-sided<br>*Only common tests should be described solely by name; describe more complex techniques in the Methods section.*
☒	☐	A description of all covariates tested
☐	☒	A description of any assumptions or corrections, such as tests of normality and adjustment for multiple comparisons
☐	☒	A full description of the statistical parameters including central tendency (e.g. means) or other basic estimates (e.g. regression coefficient) AND variation (e.g. standard deviation) or associated estimates of uncertainty (e.g. confidence intervals)
☐	☒	For null hypothesis testing, the test statistic (e.g. F, t, r) with confidence intervals, effect sizes, degrees of freedom and P value noted<br>*Give P values as exact values whenever suitable.*
☒	☐	For Bayesian analysis, information on the choice of priors and Markov chain Monte Carlo settings
☒	☐	For hierarchical and complex designs, identification of the appropriate level for tests and full reporting of outcomes
☐	☒	Estimates of effect sizes (e.g. Cohen's *d*, Pearson's *r*), indicating how they were calculated

Our web collection on statistics for biologists contains articles on many of the points above.

Software and code

Policy information about availability of computer code

Data collection	PATCHMASTER software (v2×90.2, HEKA).
Data analysis	Excel (Microsoft, WA, USA)<br>Igor Pro 6.3.7 (WaveMetrics, OR, USA)<br>R (version 4.3.1)<br>SigmaPlot 12.1 (Systat Software, CA, USA)<br>LTspice XVII (Analog Devices, Norwood, MA, USA)<br>NEURON simulator (v8.0)(https://www.neuron.yale.edu/neuron/)<br>ModelDB, #143100<br>izap.mod (The NEURON Forum, https://www.neuron.yale.edu/ftp/ted/neuron/izap.zip)<br>Zenodo (DOI: 10.5281/zenodo.15406914)

For manuscripts utilizing custom algorithms or software that are central to the research but not yet described in published literature, software must be made available to editors and reviewers. We strongly encourage code deposition in a community repository (e.g. GitHub). See the Nature Portfolio guidelines for submitting code & software for further information.

Data

Policy information about availability of data

All manuscripts must include a data availability statement. This statement should provide the following information, where applicable:

- Accession codes, unique identifiers, or web links for publicly available datasets
- A description of any restrictions on data availability
- For clinical datasets or third party data, please ensure that the statement adheres to our policy

The datasets generated during and/or analyzed during the current study are available from the corresponding author upon reasonable request. All data in graphs and Tables are summarized in Supplementary Data 1.
HEK293 cells (JCRB Cell Bank, Cat No. JCRB9068, RRID: CVCL_0045, Osaka, Japan).

Research involving human participants, their data, or biological material

Policy information about studies with human participants or human data. See also policy information about sex, gender (identity/presentation), and sexual orientation and race, ethnicity and racism.

Reporting on sex and gender	N/A
Reporting on race, ethnicity, or other socially relevant groupings	N/A
Population characteristics	N/A
Recruitment	N/A
Ethics oversight	N/A

Note that full information on the approval of the study protocol must also be provided in the manuscript.

Field-specific reporting

Please select the one below that is the best fit for your research. If you are not sure, read the appropriate sections before making your selection.

☒ Life sciences ☐ Behavioural & social sciences ☐ Ecological, evolutionary & environmental sciences

For a reference copy of the document with all sections, see nature.com/documents/nr-reporting-summary-flat.pdf

Life sciences study design

All studies must disclose on these points even when the disclosure is negative.

Sample size	The sample sizes and statistical tests were chosen based on previous studies with similar methodologies.
Data exclusions	If the electrode potential polarization was >5 mV from the initial value at the end of the recording, the data were omitted from the analysis. Data from recordings with high series resistances (>10 MΩ) were also omitted.
Replication	Transfection was confirmed by the presence or absence of evoked K+ currents and EGFP expression after whole-cell recordings were established. Cells that exhibited no clear depolarization-evoked currents and were negative for EGFP expression (i.e., non-transfected) in the same dish were used as controls. Recordings were performed from HEK293 or HEK293T cells cultured in three or more dishes.
Randomization	Experiments were performed from HEK293 cells in dishes randomly selected. Researchers select HEK293 cells without information about the transfection.
Blinding	Investigators performed whole-cell recording from HEK293 cells on the dish without information of ion channel expression. Therefore, investigators were blinded to whether cells had been successfully transfected or not at the start of experiments. After recording, transfection was checked by ion channel currents and GFP expressions.

Reporting for specific materials, systems and methods

We require information from authors about some types of materials, experimental systems and methods used in many studies. Here, indicate whether each material, system or method listed is relevant to your study. If you are not sure if a list item applies to your research, read the appropriate section before selecting a response.

Materials & experimental systems

n/a	Involved in the study
☒	☐ Antibodies
☐	☒ Eukaryotic cell lines
☒	☐ Palaeontology and archaeology
☒	☐ Animals and other organisms
☒	☐ Clinical data
☒	☐ Dual use research of concern
☒	☐ Plants

Methods

n/a	Involved in the study
☒	☐ ChIP-seq
☒	☐ Flow cytometry
☒	☐ MRI-based neuroimaging

Eukaryotic cell lines

Policy information about cell lines and Sex and Gender in Research

Cell line source(s)	HEK293 cells were purchased from JCRB Cell Bank (Cat No. JCRB9068, RRID: CVCL_0045, Osaka, Japan). HEK293T cells were provided by Dr. Satoshi Okada (Hiroshima University, Hiroshima, Japan).
Authentication	HEK293 cells: JCRB Cell Bank
Mycoplasma contamination	Contamination free was checked by JCRB Cell Bank.
Commonly misidentified lines (See ICLAC register)	*Name any commonly misidentified cell lines used in the study and provide a rationale for their use.*

Plants

Seed stocks	N/A
Novel plant genotypes	N/A
Authentication	N/A
